# Estimating the Octanol/Water Partition Coefficient for Aliphatic Organic Compounds Using Semi-Empirical Electrotopological Index

**DOI:** 10.3390/ijms12107250

**Published:** 2011-10-24

**Authors:** Erica Silva Souza, Laize Zaramello, Carlos Alberto Kuhnen, Berenice da Silva Junkes, Rosendo Augusto Yunes, Vilma Edite Fonseca Heinzen

**Affiliations:** 1 Departamento de Química, Universidade Federal de Santa Catarina, Campus Universitário, Trindade, Florianópolis, SC 88040-970, Brazil; E-Mails: ericass07@gmail.com (E.S.S.); ryunes@msn.com (R.A.Y.); 2 Departamento de Física, Universidade Federal de Santa Catarina, Campus Universitário, Trindade, Florianópolis, SC 88040-970, Brazil; E-Mails: lzaramello@gmail.com (L.Z.); kuhnen@fsc.ufsc.br (C.A.K.); 3 Instituto Federal de Educação, Ciência e Tecnologia de Santa Catarina, Avenida Mauro Ramos 950, Florianópolis, SC 88020-300, Brazil; E-Mail: berenice@ifsc.edu.br

**Keywords:** quantitative structure-property relationship, n-octanol-water partition coefficient, semi-empirical electrotopological index

## Abstract

A new possibility for estimating the octanol/water coefficient (log *P*) was investigated using only one descriptor, the semi-empirical electrotopological index (*I*_SET_). The predictability of four octanol/water partition coefficient (log *P*) calculation models was compared using a set of 131 aliphatic organic compounds from five different classes. Log *P* values were calculated employing atomic-contribution methods, as in the Ghose/Crippen approach and its later refinement, AlogP; using fragmental methods through the ClogP method; and employing an approach considering the whole molecule using topological indices with the MlogP method. The efficiency and the applicability of the *I*_SET_ in terms of calculating log *P* were demonstrated through good statistical quality (*r* > 0.99; *s* < 0.18), high internal stability and good predictive ability for an external group of compounds in the same order as the widely used models based on the fragmental method, ClogP, and the atomic contribution method, AlogP, which are among the most used methods of predicting log *P*.

## 1. Introduction

The logarithm of the molecular 1-octanol-water partition coefficient (log *P*) of compounds, which is a measure of hydrophobicity, is widely used in numerous Quantitative Structure-Activity Relationship (QSAR) models for predicting the pharmaceutical properties of molecules [1–7]. In medicinal chemistry there is continued interest in developing methods of deriving log *P* based on molecular structure. From the experimental point of view the equilibrium methods for the determination of partition coefficients are difficult or, in some cases, impossible, as in the case of instable compounds or due to impurities. Other difficulties are associated with the formation of stable emulsions after shaking or compounds which have a strong preference for one of the phases of the system. Thus, the agreement between the theoretical and experimental approaches to the determination of partition coefficients continues to be a focus of scientific interest [8]. Despite the huge amount of experimental data on the log *P* values of organic structures, this is still insufficient compared with the number of compounds for which log *P* is of interest [5]. The first method of calculating log *P* was the π-system, developed by Hansch and Fujita [9,10]. Several different methods for calculating the log *P* values from chemical structure have in common that molecules are cut into groups or atoms; summing the fragmental or single-atom contribution results, to give the final log *P* value.

The most widely used method for calculating log *P* is the fragmental method [11], which is based on the additive constitutive properties of log *P*. In the case of the atomic-contribution method [12] the atom type is used instead of a fragment. This approach was developed in an effort to attribute properties to an atom within a molecular structure and most of these methods do not use correction factors, as in the fragmental methods. The more recent approaches consider the molecule as a whole. These models attempt to make theoretical estimations of log *P*, using graph-theoretical descriptors, molecular properties or quantum-chemical descriptors to quantify log *P*, some methods incorporating the effects of the three-dimensional structure and the electronic properties of the molecule [13–22]. Several researchers have compared the predictive ability of log *P* calculation models. A review was published by Mannhold and Waterbeemd in 2001 comparing log *P* calculations obtained from different models [5].

Recently, a new topological index, called the semi-empirical electrotopological index (*I*_SET_), was developed by our research group in order to obtain a molecular descriptor not directly related to the chromatographic retention indices (RI) but based on values calculated by quantum mechanics to obtain Quantitative Structure-Property Relationship (QSPR) for different classes of organic compounds. This new approach takes into account the charges of the heteroatom and the carbon atoms attached to them through the definition of an equivalent local dipole moment [23–26].

The main goal of this study is to compare the predictive power of four log *P* calculation models and *I*_SET_ for a set of 131 aliphatic organic compounds from five different classes. The external validation of the models is performed using the cross-validation coefficient, *r*_cv_ ^2^, and seven experimental log *P* values for aliphatic alcohols are calculated, which are not included in the training sets for each model.

## 2. Methods

The QSPR study of these aliphatic organic compounds was performed with the selection of the data set, generation of molecular descriptors, simple linear regression statistical analysis and model validation techniques. The model applicability was further examined by plotting predicted data against experimental data for all of the compounds. All regression analysis was carried out using the Origin [27] and TSAR programs [28]. The statistical parameters used to test the prediction efficiency of the models obtained were the correlation coefficient (*r*), standard deviation (*s*), coefficient of determination (*r*^2^) and null hypothesis test (*F*-test). The validity of the model was tested with the cross-validation coefficient (*r*_cv_ ^2^) using “leave-one-out” in the software program TSAR 3.3 for windows [28]. A group of seven compounds, not included in the original QSPR models, was employed for the external validation.

### 2.1. Data Set and Calculation Models

The experimental Log *P* values for the organic compound groups studied herein were taken from the literature [6,7]. Theoretical values of log *P* for 131 aliphatic organic compounds were obtained using four log *P* calculation models. Log *P* calculation methods can be roughly divided into two major classes: substructure approaches which have in common that molecules are cut into groups (fragmental methods) or atoms (atomic-contribution methods) (property-based models); and whole-molecule approaches that consider the entire molecule using molecular lipophilicity potentials, topological indices or molecular properties. Atomic-contribution methods do not usually require correction factors. The almost identical methodological background of the fragmental and atomic-contribution methods indicates their interchangeability.

Log *P* values were calculated employing atomic-contribution methods as in the Ghose/Crippen approach [12] (available in the Hyperchem package [29]) or its later refinement, AlogP [30,31], and using fragmental methods such as the ClogP method [32] available in the Osiris Property Explorer package [33]. ClogP and AlogP methods are among the most prominent methods of predicting log *P*. Both methods have been implemented as part of free and commercial software programs for molecular modeling applications [29,33,34]. Values of log *P* derived from the whole-molecule approach were calculated using topological indices as in the MlogP method [35]. AlogP and MlogP are available in the VCCLAB on-line software package (ALOGPS 2.1 program) [34]. The calculated and the experimental log *P* values for 131 organic compounds in the test set are shown in Table 1. The theoretical values were then determined using the models of Ghose/Crippen, AlogP, ClogP, MlogP and the present model through the *I*_SET_ molecular descriptor. As can be seen in Table 1, some experimental log *P* values are missing, which may be related to the inherent difficulties associated with the determination of log *P* for certain compounds. However, their calculated values are included herein to allow future comparison with experimental values.

### 2.2. Semi-Empirical Electrotopological Index, I_SET_

In this study, the new descriptor, that is, the recently developed electrotopological index, *I*_SET_ [23–26], is applied to QSPR studies to predict the octanol/water partition coefficient, Log *P*, for a large amount of organic compounds, including aliphatic hydrocarbons such as alkanes and alkenes, aldehydes, ketones, esters and alcohols. This new descriptor can be quickly calculated for this series of molecules from the semi-empirical, quantum-chemical, AM1 method and correlated with the approximate numerical values attributed by the semi-empirical topological index to the primary, secondary, tertiary and quaternary carbon atoms. Thus, unifying the quantum-chemical with the topological method gives a three-dimensional picture of the atoms in the molecule [23]. It is important to note that the AM1 method gives more reliable semi-empirical charges, dipoles and bond lengths than those obtained from time-consuming, low-quality, *ab initio* methods, that is, when employing a minimal basis set in *ab initio* calculations [36]. Despite the fact that the calculated partial atomic charges may be less reliable than other molecular properties, and that different semi-empirical methods give values for the net charges with poor numerical agreement, it is important to recognize that their calculation is easy and that the values at least indicate trends in the charge density distributions in the molecules. Since many chemical reactions or physico-chemical properties are strongly dependent on local electron densities, net atomic charges and other charge-based descriptors are currently used as chemical reactivity indices [37].

For alkanes and alkenes, this correlation has allowed the creation of a new semi-empirical electrotopological index (*I*_SET_) for QSRR models [20] based on the fact that the interactions between the solute and the stationary phase are due to electrostatic and dispersive forces. This new index, *I*_SET_, is able to distinguish between the *cis*- and *trans*-isomers directly from the values of the net atomic charges of the carbon atoms that are obtained from quantum-chemical calculations. For polar molecules like aldehydes, ketones, esters and alcohols, the presence of heteroatoms like oxygen changes considerably the charge distribution of the corresponding hydrocarbons giving a partial increase in the interactions between the solute and the stationary phase. An appropriate way to calculate the *I*_SET_ was developed, which takes into account the dipole moment exhibited by these molecules and the atomic charges of the heteroatoms and the carbon atoms attached to them. By considering the stationary phase as a non-polar material, the interaction between these molecules and the stationary phase are electrostatic with a contribution from dispersive forces. These interactions slowly increase relative to the corresponding hydrocarbons. Hence, the interactions between the molecules and the stationary phase slowly increase and, clearly, this is due to the charge redistribution that occurs in the presence of the heteroatom. This charge redistribution accounts for the dipole moment of the molecules. The dispersive force between these kinds of molecules and the stationary phase includes the charge-dipole interactions and dipole-induced dipole interactions, which are weak relative to the electrostatic interactions. Thus, the dipolar charge distribution in such molecules leads to a small increase in the interactions of the solute with the stationary phase relative to hydrocarbons where the dipole moment is zero, or almost zero. Clearly, the major effects on the charge distribution due to the presence of the (oxygen) heteroatoms occur in its neighborhood and the excess charge at these atoms leads to electrostatic interactions that are stronger than the weak dispersive dipolar interactions.

For aldehydes, ketones, esters and alcohols all these factors were included in the calculation of the retention index through a small increase in the values for the atomic descriptor (named *SET*_i_) for the heteroatoms and carbon atom attached to them [24–26]. This was achieved by multiplying the *SET*_i_ values of these atoms by a function *A*_μ_ which is logarithmically dependent on the dipole moment of the molecule and the net charge at the oxygen and carbon atoms (to include both the electrostatic and dispersive interactions) that are embodied in the definition of the local dipole moment *μ*_F_ [24–26]. In this approach the dispersive dipolar interactions were included in the calculation of the retention index by multiplying the *SET*_i_ values of the heteroatoms (oxygen) and carbon atoms attached to the heteroatoms by the dipolar function *A*_μ_. That is, in this model the *I*_SET_ is calculated as in Equation 1,

(1) $$I_{\text{SET}}=\sum I_{{\text{SET}}_{\text{i}}}=\sum_{\text{i},\text{j}}\left(A_{\mu }{\mathrm{SET}}_{\text{i}}+\text{log}\;A_{\mu }{\mathrm{SET}}_{\text{j}}\right)$$

where the *SET*_i_ values are obtained through a linear relationship with the net atomic charge obtained from AM1 calculations [18–21]. In Equation 1,*A*_μ_ is logarithmically dependent on the dipole moment of the molecule, as in Equation 2:

(2) $$A_{\mu }=1+\text{log}(1+\frac{\mu }{\mu_{\text{F}}})$$

where *μ* is the calculated molecular dipole moment and *μ*_F_ is the equivalent local dipole moment which is dependent on the charges of the atoms belonging to the C-heteroatom group. In the above expression for the *I*_SET_ (Equation 1) the dipolar function *A*_μ_ is taken as the unit for the remaining carbon atoms of the molecules. The various definitions of the local dipole moment *μ*_F_ are given in previous papers concerned with the retention index of aldehydes, ketones, esters and alcohols [24–26].

For the *I*_SET_ model, the AM1 semi-empirical calculations of the net atomic charges were performed using the Hyperchem software package [29]. The initial geometries were obtained through molecular mechanics (MM+) calculations, being subsequently optimized using the AM1 method [36,38], employing the Polak-Ribiere algorithm and gradient minimization techniques with a convergence limit of 0.0001 and RMS gradient of 0.0001 kcal (A mol)^−1^. Mulliken population analysis was employed to obtain the net atomic charge of the carbon atoms and oxygen atoms. The net atomic charge (*Q*_i_) is obtained from the difference between the electronic charge of the isolated atom (Z) and the calculated charge of the bound atom (*q*_i_), that is, *Q*_i_ = Z − *q*_i_. The *SET*_i_ values for each atom are obtained from Equation 2 using the AM1 net atomic charges (*Q*_i_). Employing AM1 calculations these quantities are more easily obtained for a large number of molecules of reasonable size compared with those obtained when employing a minimal basis set in *ab initio* calculations [36]. Despite of the usually limited quantitative accuracy of semi-empirical methods the computational efficiency available nowadays [35] enables electronic properties of a large number of molecules to be obtained in a reasonable amount of time, and computational time is an important feature when developing models of quantitative structure-activity relationships (QSAR)[37].

## 3. Results and Discussion

The 3-hexanone molecule represented in the graph below is taken as an example of the *I*_SET_ calculation using the present approach. The net atomic charges and *SET*_i_ values are given in Table II of the reference 24.

**Figure:**
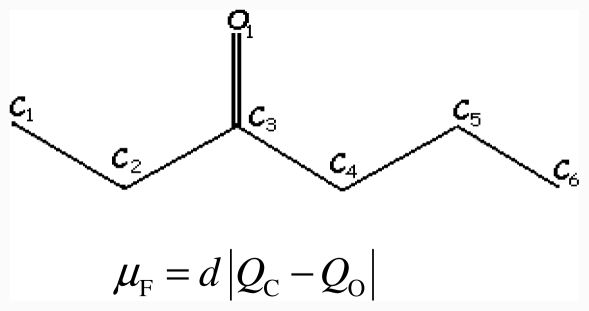


(3) $$\mu_{\text{F}}=d|Q_{\text{C}}-Q_{\text{O}}|$$

*μ*_F_ = 1.2342 |0.224 − [−0.288]| = 0.6319

*A*_μ_ = 1 + log[1 + (2.6790/0.63191)] = 1.7193

*I*_SETO1_ = (=O) = *A*_μ_*SET*_O1_ *+* log *A*_μ_*SET*_C3_ = 1.9507 + log 0.3899 = 1.5416

*I*_SETC1_ = (−CH_3_) = *SET*_C1_ *+* log *SET*_C2_ = 0.9892 + log 0.9998 = 0.9891

*I*_SETC2_ = (−CH_2_−) = *SET*_C2_ *+* log *SET*_C1_*+* log *A*_μ_*SET*_C3_ = 0.9998 + log 0.9892 + log 0.3899 = 0.5860

*I*_SETC3_ = (>C<) = *A*_μ_*SET*_C3_ *+* log *SET*_C2_ *+* log *A*_μ_*SET*_O1_ + log *SET*_C4_ = 0.3899 + log 0.9998 + log 1.9507 + log 0.9998 = 0.6799

*I*_SETC4_ = (−CH_2_−) = *SET*_C4_ *+* log *A*_μ_*SET*_C3_ *+* log *SET*_C5_ *=* 0.9998 + log 0.3899 + log 0.8988 = 0.5444

*I*_SETC5_ = (−CH_2_−) = *SET*_C5_ *+* log *SET*_C4_ *+* log *SET*_C6_ = 0.8988 + log 0.9998 + log 0.9998 = 0.8986

*I*_SETC6_ = (−CH_3_) = *SET*_C6_ *+* log *SET*_C5_ = 0.9998 + log 0.8988 = 0.9535

*I*_SET_ = 1.5416 + 0.9891 + 0.5860 + 0.6799 + 0.5444 + 0.8986 + 0.9535 = 6.1931

The results obtained in the statistical analysis of the single linear regression between experimental and calculated Log *P* values using *I*_SET_ are shown in Table 2 for each class of compounds studied. They indicate that the theoretical partition coefficients calculated using the *I*_SET_ method give good agreement with the experimental partition coefficients. The QSPR models obtained with *I*_SET_ showed high values for the correlation coefficient (*r* > 0.99), and the leave-one-out cross-validation demonstrate that the final models are statistically significant and reliable (*r*_cv_ ^2^ > 0.98). As can be observed, this model explains more than 99% of the variance in the experimental values for this set of compounds. Among the various classes of compounds the best results obtained with the *I*_SET_ method are for hydrocarbons (Table 2), which is related to the fact that the present model was developed initially for this class of organic compounds. Values of *r* = 0.9986 and *s* = 0.10 were obtained for hydrocarbons, which are the lowest values considering the other four models.

The present results can be compared with those recently published for a new approach based on the Kovats retention indices, which uses multiple linear regressions [7], where reportedly for 37 hydrocarbons *s* = 0.46, for 11 aldehydes *s* = 0.27, for 27 alcohols *s* = 0.32 and for 13 esters *s* = 0.17. As can be seen in Table 2, the lowest standard deviation was obtained for the aldehydes correlation (*s* = 0.05) and for alcohols the correlation was greater (*s* = 0.18). The range of standard deviations obtained verifies the applicability of the present approach to different classes of organic compounds. For alcohols, the earlier approach of Duchowicz *et al*. [6], based on the concept of flexible topological descriptors and on the optimization of correlation weights of local graphic invariants, is applied to model the octanol/water partition coefficient of a representative set of 62 alcohols, resulting in a satisfactory prediction with a standard deviation of 0.22. Recently, Liu *et al*. [39] carried out a QSPR study to predict the log *P* for 58 aliphatic alcohols using novel molecular indices based on graph theory, by dividing the molecular structure into substructures obtaining models with good stability and robustness, and values predicted using the multiple linear regression method are close to the experimental values (*r* = 0.9959 and *s* = 0.15). The above results show the reliability of the present model calculation based on the semi-empirical calculation of atomic charges and local dipole moments using only one descriptor, *I*_SET_.

The statistical analysis for the predictive ability of four log *P* calculation models and *I*_SET_ for a set of 131 aliphatic organic compounds from five different classes are summarized in Table 2. The AlogP method gives a stable performance for all classes of organic compounds tested, with much less variability in the statistical quality of results among different subclasses (*r* > 0.98 and *s* < 0.22). The ClogP method offers good predictability (*r* > 0.99 and *s* < 0.17), giving larger deviations only in the case of ketones (*r* = 0.955; *s* = 0.40). The MlogP and Ghose/Crippen methods have much larger deviations (*r* > 0.974 and *s* < 0.39) in comparison with the other methods.

The experimental and predicted log *P* values using *I*_SET_ and the other four models (and the respective deviations) for an external group of alcohols are shown in Table 3. The Ghose/Crippen method and its refinement AlogP shows appreciable deviations for 1-undecanol and 4,4-dimethyl-1-pentanol, respectively, whereas the ClogP values are greater for branched alcohols. For the three last branched alcohols in Table 3 the whole molecule approach MLogP, which employs an MLR with final regression equation involving 13 parameters, gives the same value for Log *P*, being unable to distinguish the structural differences between these branched alcohols. The average standard deviation of calculated Log *P* for the seven alcohols of Table 3 using the *I*_SET_ model is 0.15, whereas for the Ghose/Crippen method it is 0.34. The AlogP method, which is applicable to most neutral organic compounds and selective charged compounds, shows an average standard deviation of 0.26. In contrast, the ClogP method, which uses a large number of parameters and correction factors, results in a standard deviation of 0.17, while for the whole molecule approach the value is 0.24. These results demonstrate that the predictability of the present model for polar aliphatic organic compounds has the same pattern of accuracy as the widely used ClogP model.

The predictive ability of a QSPR model can be estimated using an external test set of compounds that has not been used for building the model. According to Tropsha and Golbraikh [40] a high value of cross-validated *r*^2^ (*q*^2^) alone is insufficient criterion for a QSAR model to be considered highly predictive, and the use of an external set of compounds for the model validation is always necessary. The authors’ state that the correlation coefficient, *r*, between the predicted and observed activities of compounds from an external test set should be close to 1 [40,41]. Following these authors, we considered seven compounds not included in the original model (Table 3) plotting observed *vs.* predicted log *P* values obtaining *Y* = 1.0273*X* − 0.1223 with *r*^2^ = 0.9858 and *Y* = 0.9893*X* (with the intercept set to 0) with *r*^2^ = 0.9842. Predicted *vs.* observed log *P* values, *Y* = 0.9596*X* + 0.1557 with *r*^2^ = 0.9858 and *Y* = 1.008*X* with *r*^2^ = 0.9828 were plotted. The QSPR model has a value of cross-validated (using leave-one-out), *r*_cv_ ^2^ = 0.9870 showing that the model has high predictive power.

## 4. Conclusions

The efficiency and the applicability of the descriptor *I*_SET_ in terms of predicting log *P* using the quantitative structure-activity relationship (QSPR) were demonstrated through the good statistical quality and high internal stability obtained for the studied classes of compounds as well as the good predictive ability for the external group of compounds. The *I*_SET_ model also has the advantage of simplicity, using only one descriptor, and it has statistical quality of the same order as the widely used models based on the fragmental method, ClogP, and the atomic-contribution method, AlogP. The quality of the results obtained can be considered appropriate for the development of QSPR models for other compounds in the future.

## Figures and Tables

**Table 1:** Semi-Empirical Electrotopological Indices (*I*_SET_), calculated values for Log *P* using Atomic-Contribution Methods (Ghose/Crippen and AlogP), Fragmental Method (ClogP), Topological indices (MlogP and *I*_SET_) and experimental Log *P* values (Log *P*_exp_) for the studied set of compounds.

No.	Class of compounds	*I*_SET_	*I*_SET_ Log *P*	Ghose/Crippen Log *P*	AlogP	ClogP	MlogP	Log *P*_exp_
**Hydrocarbon**								
01	Ethane	1.9981	1.88	1.30	1.28	1.38	1.76	1.81
02	Propane	2.8148	2.40	1.69	1.74	1.84	2.28	2.36
03	*N*-Butane	3.6343	2.91	2.09	2.20	2.31	2.73	2.89
04	*N*-Pentane	4.4457	3.43	2.49	2.65	2.77	3.14	3.39
05	*N*-Hexane	5.2622	3.95	2.88	3.11	3.23	3.52	4.00
06	*N*-Heptane	6.0787	4.46	3.28	3.57	3.70	3.87	4.50
07	*N*-Octane	6.8952	4.98	3.67	4.02	4.16	4.20	5.15
08	*N*-Nonane	7.7117	5.49	4.07	4.48	4.63	4.52	5.65
09	*N*-Decane	8.5282	6.01	4.47	4.93	5.09	4.82	6.25
10	*N*-Undecane	9.3447	6.53	4.86	5.39	5.55	5.11	6.54
11	*N*-Dodecane	10.1612	7.04	5.26	5.85	6.02	5.40	6.80
12	*N*-Tridecane	10.9777	7.56	5.66	6.30	6.48	5.67	7.50
13	*N*-Tetradecane	11.7942	8.08	6.05	6.76	6.95	5.93	8.00
14	2-Methylpropane	3.5421	2.86	2.02	1.99	2.18	2.73	2.76
15	3-Methylheptane	6.7641	4.89	3.61	3.36	4.04	3.87	
16	2.4-Dimethylpentane	5.8455	4.31	3.15	3.16	3.45	3.87	
17	Ethene	2.0294	1.20	1.13	0.95	1.15	0.70	1.13
18	Propene	2.8082	1.74	1.48	1.35	1.55	1.22	1.77
19	1-Butene	3.5848	2.28	1.87	1.81	2.01	1.67	2.40
20	1-Pentene	4.3996	2.84	2.27	2.26	2.48	2.08	2.80
21	1-Hexene	5.2140	3.40	2.67	2.72	2.94	2.46	3.40
22	1-Heptene	6.0305	3.96	3.06	3.17	3.40	2.81	3.99
23	1-Octene	6.8606	4.53	3.46	3.63	3.87	3.15	4.57
24	*E-*2-Octene	6.7939	4.49	3.41	3.58	3.80	3.15	4.44
25	2-Ethylhexene	6.5614	4.33	3.22	3.57	3.35	3.15	4.31
**Aldehyde**								
01	Acetaldehyde	3.3967	−0.23	−0.58	−0.18	0.43	−0.32	−0.22
02	Propionaldehyde	4.1866	0.27	0.05	0.48	0.89	0.20	0.30
03	Butyraldehyde	5.0052	0.79	0.44	0.94	1.36	0.65	0.83
04	Hexanal	6.6508	1.85	1.24	1.85	2.28	1.44	1.89
05	Heptanal	7.4709	2.38	1.63	2.31	2.75	1.79	2.42
06	Octanal	8.2859	2.89	2.03	2.77	3.21	3.04	2.90
07	2-Methyl-1-Propanal	5.6519	0.73	0.61	0.95	1.23	0.65	0.77
08	*E-*2-Butenal	3.8057	0.60	0.52	0.92	1.00	0.55	0.52
09	*E-*2-Hexenal	5.4466	1.68	1.32	1.83	1.93	1.34	1.58
**Ketone**								
01	Acetone	4.0158	−0.08	0.38	−0.24	0.74	0.20	−0.24
02	2-Butanone	4.5952	0.30	1.01	0.42	1.21	0.65	0.29
03	2-Pentanone	5.3987	0.84	1.40	0.88	1.67	1.06	0.91
04	2-Hexanone	6.1987	1.38	1.80	1.34	2.14	1.44	1.38
05	2-Heptanone	7.0080	1.92	2.20	1.79	2.60	1.79	1.98
06	2-Octanone	7.8306	2.48	2.59	2.25	3.06	2.13	2.37
07	2-Nonanone	8.6458	3.02	2.99	2.70	3.53	3.36	3.14
08	2-Decanone	9.4583	3.57	3.39	3.16	3.99	3.66	3.73
09	2-Undecanone	10.2706	4.11	3.78	3.62	4.46	3.95	4.09
10	2-Dodecanone	11.0872	4.66	4.18	4.07	4.92	4.23	4.55
11	3-Pentanone	5.3900	0.84	1.64	1.09	1.67	1.06	0.99
12	3-Methyl-2-Butanone	5.2258	0.73	1.57	0.88	1.55	1.06	0.84
13	4-Methyl-2-Pentanone	6.0484	1.28	1.73	1.13	2.01	1.44	1.31
14	5-Nonanone	8.5885	2.98	3.22	2.91	3.53	2.45	2.88
15	3-Hexanone	6.1931	1.37	2.03	1.55	2.14	1.44	1.45
16	2.2 -Dimethyl-3 Butanone	5.8039	1.11	2.24	1.30	2.06	1.44	1.20
17	5-Methyl-2-Hexanone	6.8815	1.84	2.13	1.59	2.48	1.79	1.88
18	5-Methyl-2-Octanone	8.5182	2.94	2.92	2.50	3.40	2.45	2.92
19	2.2.4.4-Tretramethyl-3-3-Pentanone	7.7789	2.44	4.09	2.85	2.05	2.45	3.00
20	3-Methyl-2-Pentanone	6.0746	1.30	1.97	1.34	2.01	1.44	
21	4-Methyl-3-Pentanone	6.0227	1.26	2.20	1.55	2.01	1.44	
22	4-Heptanone	7.0130	1.93	2.43	2.00	2.60	1.79	
23	2.4-Dimethyl-3-Pentanone	6.6629	1.69	2.76	2.02	2.35	1.79	
**Ester**								
01	Methyl Acetate	5.2056	0.20	−0.14	0.02	0.48	0.13	0.18
02	Ethyl Acetate	5.9566	0.72	0.21	0.37	0.91	0.59	0.73
03	2-Methylbutyl Acetate	8.1580	2.23	1.47	1.67	2.18	1.73	2.29
04	Propyl Acetate	6.8215	1.31	0.67	0.89	1.38	1.00	1.24
05	Butyl Acetate	7.6480	1.88	1.07	1.35	1.84	1.37	1.82
06	3-Methylbutyl Acetate	8.1012	2.19	1.40	1.60	2.18	1.73	2.25
07	Propyl Butyrate	8.3084	2.34	1.70	2.02	2.31	1.73	2.15
08	Methyl Propionate	5.9612	0.72	0.49	0.69	0.94	0.59	0.82
09	Propyl Formate	6.0387	0.77	0.47	0.85	1.11	0.59	0.83
10	Isobutyl Isobutyrate	8.5664	2.51	2.27	2.34	2.52	2.06	2.48
11	Isopentyl Isovalerate	9.9907	3.50	2.76	2.89	3.45	2.68	3.62
12	Methyl Butyrate	6.7703	1.27	0.89	1.14	1.41	1.00	1.29
13	Methyl Isopentanoate	7.2346	1.60	1.22	1.40	1.75	1.37	1.82
14	Methyl Decanoate	11.7131	4.55	3.37	3.88	4.19	3.88	4.41
15	Ethyl Formate	5.21385	0.20	0.0	0.32	0.64	0.13	
16	Isopropyl Acetate	6.3210	0.97	0.62	0.75	1.32	1.00	
17	Isobutyl Acetate	4.2872	1.69	1.08	1.21	1.72	1.37	
18	Ethyl Butyrate	7.5262	1.80	1.23	1.49	1.84	1.37	
19	Ethyl Valerate	8.3037	2.33	1.63	1.95	2.31	1.73	
20	Ethyl Hexanoate	9.1100	2.89	2.02	2.40	2.77	2.06	
21	Ethyl Heptanoate	9.9322	3.46	2.42	2.86	3.23	2.38	
22	Ethyl Octanoate	10.7424	4.02	2.82	3.32	3.7	3.59	
23	Ethyl Nonanoate	11.5522	4.58	3.21	3.77	4.16	3.88	
24	Ethyl Decanoate	12.3802	5.15	3.61	4.23	4.43	4.16	
**Alcohol**								
01	Ethanol	5.0258	−0.03	0.08	−0.01	0.43	−0.17	−0.31
02	1-Propanol	5.8387	0.48	0.55	0.51	0.89	0.35	0.34
03	1-Butanol	6.6371	0.99	0.94	0.97	1.35	0.80	0.84
04	1-Pentanol	7.4533	1.51	1.34	1.43	1.82	1.21	1.40
05	1-hexanol	8.2626	2.03	1.73	1.88	2.28	1.59	2.03
06	1-Heptanol	9.0808	2.55	2.13	2.34	2.74	1.94	2.34
07	1-Octanol	9.8913	3.07	2.53	2.80	3.21	3.19	3.15
08	1-Nonanol	10.7101	3.60	2.92	3.25	3.67	3.50	3.57
09	1-Decanol	11.5199	4.11	3.32	3.71	4.14	3.81	4.01
10	1-Dodecanol	13.1499	5.15	4.11	4.62	5.07	4.38	5.13
11	1-Tetradecanol	14.7791	6.20	4.91	5.53	5.99	4.91	6.11
12	1-Pentadecanol	15.5986	6.72	5.30	5.99	6.46	5.17	6.64
13	1-Hexadecanol	16.4091	7.24	5.70	6.45	6.92	5.42	7.17
14	1-octadecanol	18.039	8.28	6.49	7.36	7.85	5.90	8.22
15	2-Propanol	5.1764	0.061	0.49	0.37	0.83	0.35	0.05
16	2-Butanol	6.1384	0.67	0.96	0.89	1.29	0.80	0.61
17	2-pentanol	6.8713	1.14	1.36	1.35	1.76	1.21	1.14
18	2-Hexanol	7.6936	1.67	1.75	1.80	2.22	1.59	1.61
19	2-Heptanol	8.5136	2.19	2.15	2.26	2.68	1.94	2.31
20	2-Octanol	9.3313	2.71	2.54	2.72	3.15	2.27	2.84
21	2-Nonanol	10.1490	3.24	2.94	3.17	3.61	3.50	3.36
22	3-Pentanol	6.9241	1.17	1.43	1.42	1.76	1.21	1.14
23	3-hexanol	7.7334	1.69	1.82	1.87	2.22	1.59	1.61
24	3-Heptanol	8.5339	2.20	2.22	2.33	2.68	1.94	2.31
25	3-Nonanol	10.1594	3.24	3.01	3.24	3.61	2.59	3.36
26	4-Heptanol	8.4277	2.14	2.22	2.33	2.68	1.94	2.31
27	4-Nonanol	10.0707	3.19	3.01	3.24	3.61	2.59	3.36
28	5-Nonanol	10.0579	3.18	3.01	3.24	3.61	2.59	3.36
29	2-Methyl-1-propanol	6.7118	1.04	1.34	0.83	1.23	0.80	0.65
30	2-Methyl-1-pentanol	8.0889	1.92	1.74	1.75	2.16	1.59	1.78
31	2-Methyl-2-propanol	5.6439	0.36	0.57	0.57	0.98	0.80	0.37
32	2-Methyl-2-butanol	6.4088	0.85	1.04	1.10	1.44	1.21	0.89
33	2-Methyl-2-pentanol	7.2184	1.36	1.43	1.55	1.91	1.59	1.39
34	2-Methyl-2-hexanol	8.0185	1.87	1.83	2.01	2.37	1.94	1.84
35	2-Methyl-3-pentanol	7.6238	1.62	1.83	1.74	2.10	1.59	1.67
36	3-Methyl-1-butanol	7.3289	1.43	1.27	1.22	1.69	1.21	1.42
37	3-Methyl-2-butanol	6.7223	1.05	1.36	1.21	1.63	1.21	1.14
38	3-Methyl-2-pentanol	7.5616	1.58	1.76	1.67	2.10	1.59	1.67
39	3-Methyl-3-pentanol	7.1923	1.35	1.51	1.62	1.91	1.59	1.39
40	3-Methyl-3-hexanol	7.9993	1.86	1.90	2.08	2.37	1.94	1.87
41	4-Methyl-1-pentanol	8.1457	1.96	1.67	1.68	2.16	1.59	1.78
42	4-Methyl-2-pentanol	7.5971	1.60	1.69	1.60	2.10	1.59	1.67
43	5-Methyl-2-hexanol	8.4042	2.12	2.08	2.06	2.56	1.94	2.19
44	2-Ethyl-1-butanol	8.0637	1.90	1.74	1.75	2.16	1.59	1.78
45	2-Ethyl-1-hexanol	9.6883	2.94	2.53	2.66	3.08	2.27	2.84
46	3-Ethyl-3-pentanol	7.9941	1.86	1.97	2.14	2.37	1.94	1.87
47	2.2-Dimethyl-1-propanol	6.8319	1.11	1.45	1.11	1.74	1.21	1.36
48	2.2-Dimethyl-1-butanol	7.6225	1.62	1.85	1.56	2.21	1.59	1.57
49	2.2-Dimethyl-1-pentanol	8.0200	1.87	2.25	2.02	2.67	1.94	2.39
50	2.2-Dimethyl-3-pentanol	7.9220	1.81	2.34	2.01	2.61	1.94	2.27
51	2.3-Dimethyl-1-butanol	7.7752	1.72	1.68	1.54	2.03	1.59	1.17
52	2.3-Dimethyl-2-butanol	7.1113	1.29	1.44	1.42	1.78	1.59	1.17
53	2.3-Dimethyl-2-pentanol	7.9254	1.81	1.84	1.87	2.25	1.94	2.27
54	2.4-Dimethyl-1-pentanol	8.7738	2.36	2.07	2.00	2.50	1.94	2.19
55	2.4-Dimethyl-2-pentanol	7.7712	1.727	1.76	1.80	2.25	1.94	1.67
56	2.4-Dimethyl-3-pentanol	8.0997	1.93	2.23	2.05	2.44	1.94	2.31
57	2.6-Dimethyl-4-heptanol	9.8577	3.05	2.88	2.83	3.36	2.59	3.13
58	3.3-Dimethyl-1-butanol	7.3456	1.44	1.71	1.43	2.21	1.59	1.57
59	3.3-Dimethyl-2-butanol	7.2531	1.38	1.87	1.49	2.15	1.59	1.19
60	2.2.3-Trimethyl-3-pentanol	8.2383	2.01	2.41	2.21	2.76	2.27	1.99

**Table 2:** The coefficients a and b (*Y* = a + b*X*) and statistical parameters (*r*^2^, *r*, *F*, *s*, *r*_cv_ ^2^) for linear regressions between experimental and calculated Log *P* values using different methods (Ghose/Crippen Log *P*, AlogP, MlogP, ClogP, and *I*_SET_ Log *P*) for each class of compounds studied (according to Table 1).

Class	Method	*N*	a	b	*r*^2^	*r*	*F*	*s*	*r*_cv_^2^
Hydrocarbon	Ghose/Crippen Log *P*	23	−0.0740	1.3559	0.9925	0.9962	2760.8	0.1694	0.9907
	AlogP	23	0.3080	1.1554	0.9952	0.9976	4345.4	0.1352	0.9940
	ClogP	23	0.1451	1.1513	0.9923	0.9961	2694.0	0.1715	0.9904
	MlogP	23	−0.0923	1.2953	0.9565	0.9780	462.2	0.4066	0.9494
	*I*_SET_ Log *P*	23	0.0039	0.9997	0.9971	0.9986	7289	0.1045	0.9964
Alcohol	Ghose/Crippen Log *P*	60	−0.6651	1.3623	0.9822	0.9911	3202.8	0.2196	0.9813
	AlogP	60	−0.3038	1.1600	0.9897	0.9949	5592.7	0.1668	0.9893
	ClogP	60	−0.7966	1.1550	0.9914	0.9957	6651.4	0.1531	0.9910
	MlogP	60	−0.4666	1.3344	0.9611	0.9803	1431.6	0.3249	0.9561
	*I*_SET_ Log *P*	60	3,2482	0,6394	0.9876	0.9938	4612.6	0.1835	0.9870
Aldehyde	Ghose/Crippen Log *P*	9	0.2243	1.2357	0.9539	0.9767	145.0	0.2318	0.9134
	AlogP	9	−0.2236	1.0954	0.9789	0.9894	324.6	0.1611	0.9613
	ClogP	9	−0.6533	1.1187	0.9979	0.9990	3388.8	0.0503	0.9966
	MlogP	9	0.1668	1.0159	0.9489	0.9741	130.0	0.2566	0.8469
	*I*_SET_ Log *P*	9	0.0016	1.0014	0.9972	0.9986	2525.9	0.0583	0.9961
Ketone	Ghose/Crippen Log *P*	19	−0.8484	1.2097	0.9188	0.9585	192.3	0.3861	0.8867
	AlogP	19	−0.1299	1.1494	0.9862	0.9931	1213.4	0.1593	0.9829
	ClogP	19	−0.8479	1.1132	0.9115	0.9547	175.1	0.4031	0.8974
	MlogP	19	−0.2586	1.1454	0.9694	0.9846	538.8	0.2370	0.9622
	*I*_SET_ Log *P*	19	−2.7182	0.6693	0.9864	0.9932	1229.7	0.1582	0.9831
Ester	Ghose/Crippen Log *P*	14	0.3894	1.1472	0.9688	0.9843	372.9	0.2124	0.9573
	AlogP	14	0.1815	1.1080	0.9681	0.9839	364.7	0.2147	0.9590
	ClogP	14	−0.3054	1.1334	0.9943	0.9971	2076.6	0.0912	0.9928
	MlogP	14	0.1370	1.1742	0.9851	0.9925	791.6	0.1470	0.9630
	*I*_SET_ Log *P*	14	−3.1575	0.6587	0.9903	0.9951	1222.9	0.1186	0.9838

a = intercept; b = slope; *r*^2^ = coefficient of determination; *r* = correlation coefficient; *s* = standard deviation; *r*_cv_ ^2^ = cross-validation coefficient; *F* = null hypothesis test (*F*-test).

**Table 3:** Difference between experimental and predicted Log *P* (ΔLog *P*) using *I*_SET_ and the different methods studied (Ghose/Crippen, AlogP, MlogP, ClogP) for external group of alcohols.

No.	Compounds	Log *P*_exp_	*I*_SET_	Δ*I*_SET_ Log *P*	ΔGhose/Crippen Log *P*	ΔAlogP	ΔClogP	ΔMlogP
01	1-Undecanol	4.42	12.3394	−0.22	0.7	0.26	−0.18	0.32
02	2-Undecanol	4.42	11.7816	0.14	0.6	0.33	−0.12	0.32
03	4-Octanol	2.68	9.2504	0.02	0.06	−0.1	−0.47	0.41
04	2-Methyl-1-butanol	1.14	7.2774	−0.26	−0.2	−0.15	−0.55	−0.07
05	2-Methyl-3-hexanol	2.19	8.2667	0.16	−0.04	0	−0.37	0.25
06	2.3-Dimethyl-3-pentanol	1.67	7.78	−0.05	−0.24	−0.27	−0.58	−0.27
07	4.4-Dimethyl-1-pentanol	2.39	8.6815	0.09	0.29	0.51	−0.28	0.45
